# Adaptive weighted dual MAML: Proposing a novel method for the automated diagnosis of partial sleep deprivation

**DOI:** 10.1371/journal.pone.0325288

**Published:** 2025-06-13

**Authors:** Soraya Khanmohmmadi, Toktam Khatibi, Golnaz Tajeddin, Elham Akhondzadeh, Amir Shojaee

**Affiliations:** 1 Faculty of Industrial and Systems Engineering, Tarbiat Modares University, Tehran, Iran; 2 Faculty of Medical Science, Tarbiat Modares University, Tehran, Iran; Firat University, TÜRKIYE

## Abstract

**Introduction:**

Sleep disorders significantly disrupt normal sleep patterns and pose serious health risks. Traditional diagnostic methods, such as questionnaires and polysomnography, often require extensive time and are susceptible to errors. This highlights the need for automated detection systems to enhance diagnostic efficiency. This study proposes a novel method for the automated diagnosis of partial sleep deprivation utilizing electroencephalogram (EEG) signals.

**Materials and methods:**

We utilized time-frequency images obtained from continuous wavelet transforms applied to two EEG channels for the automated diagnosis of sleep disorders. Although convolutional neural networks (CNNs) are commonly used for detecting these conditions, their performance is inadequate when applied to our heterogeneous and limited-scale EEG data. To overcome these limitations, we developed a Few-Shot Learning-based Model-Agnostic Meta-Learning (FSL-based MAML) approach aimed at improving classification accuracy and generalization abilities. Our method, Adaptive Weighted Dual MAML, combines two base models—a ResNet and a CNN-Transformer—within the MAML framework, which leverages multi-shot tasks to improve the EEG signal classification,

**Results:**

Our findings demonstrated that the FSL-based MAML method, with a combined base model, achieves an average classification accuracy of 99% and an F1 score of 99%. Additionally, the proposed model achieved a more stable range of evaluation metrics, resulting in reduced performance fluctuations across tasks compared to the conventional MAML. This indicates stronger robustness and improved generalization to unseen tasks,

**Conclusions:**

The results confirm the efficacy of our proposed approach as a robust solution for diagnosing partial sleep deprivation with enhanced accuracy and efficiency in an automated manner. This model provides a groundwork for addressing various sleep disorders through advanced EEG analysis techniques.

## 1. Introduction

Mental disorders refer to disruptions in psychological, biological, or developmental mechanisms. These conditions often manifest as severe anxiety or difficulties in conducting essential tasks in various aspects of life [1]. According to the World Health Organization, anxiety, depression, and sleep disorders are prevalent mental disorders [2]. These conditions often coexist and are interconnected, leading to heightened challenges in their identification and diagnosis [2]. Sleep disorders are caused by inadequate sleep, age, illness, nonstandard working hours, and environmental conditions. They decrease physical and cognitive performance while also increasing the risk of stroke, diabetes, obesity, cancer, and cardiovascular disease [3]. These consequences impose heavy costs on society [4]. These costs significantly impact health, welfare, safety, and productivity. It is crucial to recognize the economic implications associated with sleep disorders to effectively allocate resources toward addressing these issues [5]. The costs incurred due to sleep disorders can be categorized into two fundamental areas: financial and nonfinancial [6]. The financial costs include expenses related to healthcare, productivity losses, informal care, nonmedical costs, and deadweight loss. On the other hand, the nonfinancial costs encompass the negative impact on individuals’ quality of life, leading to feelings of sadness and difficulty [6]. Recognizing and acknowledging these costs can lead to appropriate actions being taken to mitigate the effects of sleep disorders and enhance welfare [5].

Various sleep disorders can be identified through the utilization of polysomnography (PSG), i.e., the analysis of electrical biological signals, such as the electroencephalogram (EEG), the electromyograph (EMG), the electrooculography (EOG), and the electrocardiograph (ECG) [7]. EEG is the fundamental modality [8], and a powerful tool for diagnosing sleep disorders [8,9]. EEG is a technology that can be implemented anywhere [10], is portable, non-invasive, cost-effective, and has high resolution [11]. Given these advantages, the EEG modality is proposed as a systematic and practical physiological technique for diagnosing sleep disorders in this study. Since the information in two-dimensional data has the advantage that more information can be extracted compared to one-dimensional data [12], this study suggests using 2D frequency-time images obtained by performing a continuous wavelet transform (CWT) from EEG signals.

Visual interpretation of EEG is a laborious and time-consuming process that requires extensive training for doctors to acquire the necessary skills. Additionally, there is a limited level of agreement among experts when interpreting EEG signals, as shown by the low inter-rater agreement [13]. Therefore, to save time for physicians and make more accurate decisions, it is necessary to develop machine learning algorithm-based automated methods for analyzing EEG data [13].

Deep Learning-based methods, as a subset of machine learning methods, have the advantage of automatically conducting feature extraction. Manual feature extraction methods need domain knowledge, often leading to the loss of essential information [14]. Therefore, deep learning models are a suitable option for effectively extracting features to analyze frequency-time images obtained from EEG signals. However, it is essential to note that EEG signals are considered challenging data due to two limitations. These limitations may make it difficult to develop a DL-based model for automated feature extraction from images obtained from EEG signals. Firstly, in most cases, there are only a few samples in the EEG signals dataset. Secondly, EEG signals are heterogeneous, varying over time due to environmental, experimental, physiological, and psychological factors [15,16].

DL-based models depend on a significant number of labeled EEG training samples to achieve satisfactory generalization capability. Additionally, these models assume that the EEG signals in the test dataset were observed during the training phase. Nevertheless, it is possible for test EEG signals to not align with any class within the training set [17]. The diversity in EEG data from different cases makes it difficult for a model trained on existing cases to be suitable for a new case [16]. Therefore, it is crucial to develop generalizable models trained on existing cases that can also be appropriate for a new case.

Therefore, in this study, we propose and design a novel method for the automated diagnosis of partial sleep deprivation from electroencephalogram (EEG) signals. For this purpose, we utilize frequency-time images generated from continuous wavelet transform obtained from only two EEG channels for the automated detection of sleep disorders. To enhance the performance of deep neural networks trained on images extracted and generated from EEG signals, we designed a meta-learning model to address the challenges posed by small-scale and heterogeneous EEG signals and to improve the generalization capability of the model.

Meta-learning employs a learning strategy capable of rapidly acquiring new tasks [18], even with limited training samples [19]. Recent studies on EEG signal classification have brought attention to the challenges presented by inter-subject variability and limited labeled data. Despite the various approaches taken in these studies, their common goal is to enhance generalization across different subjects and tasks, showcasing the potential of meta-learning techniques in addressing the constraints of conventional machine learning models for EEG-based applications [20–22]. Most meta-learning approaches focus primarily on few-shot learning (FSL) [19]. These approaches can be classified into three groups: optimization-based, model-based, and metric-based methods [23].

In this study, we designed an MAML-based framework, an optimization-based meta-learning algorithm, to analyze the EEG signals of individuals experiencing partial sleep deprivation (PSD). MAML was designed to acquire and adjust the parameter initialization of the base model, enabling it to perform effectively on a new task with only a few gradient steps [24]. However, relying on a single base model may limit the generalization capability. Since different models capture various aspects of the data, we propose a novel approach called Adaptive Weighted Dual MAML, which adaptively combines two base models in MAML through a weighted mechanism to enhance performance. This allows the system to leverage diverse feature representations and balance their strengths. The main contributions of this paper are summarized in the following items, listed in order of importance:

• **Designing a Novel FSL-Based MAML Method for PSD Diagnosis from EEG signals**: This study presents a novel approach leveraging Few-Shot Learning (FSL) integrated with Model-Agnostic Meta-Learning (MAML) for the diagnosis of partial sleep deprivation (PSD) from EEG-derived images.
• **A hybrid of a CNN-Transformer and ResNet as the base model in MAML:** The hybrid model offers superior feature extraction capabilities. Our framework combines these architectures to create a robust, consistent, and highly performant solution for PSD detection, surpassing their individual implementations. Through adaptive weighting, our approach dynamically adjusts the contribution of each model during training, enhancing robustness and improving generalization to new tasks.
• **Enhanced Image Generation Using Continuous Wavelet Transform**: EEG signals are converted into images using Continuous Wavelet Transform, allowing for the training of numerous deep learning models that are specifically optimized for image data. This two-dimensional representation captures more comprehensive information than traditional one-dimensional data.
• **Simplification of PSD Diagnosis with Minimal Number of EEG Channels**: The diagnostic process for PSD is streamlined by utilizing only two EEG channels, effectively reducing the computational demands and hardware requirements while maintaining diagnostic efficacy.

## 2. Materials and methods

**Fig 1:**
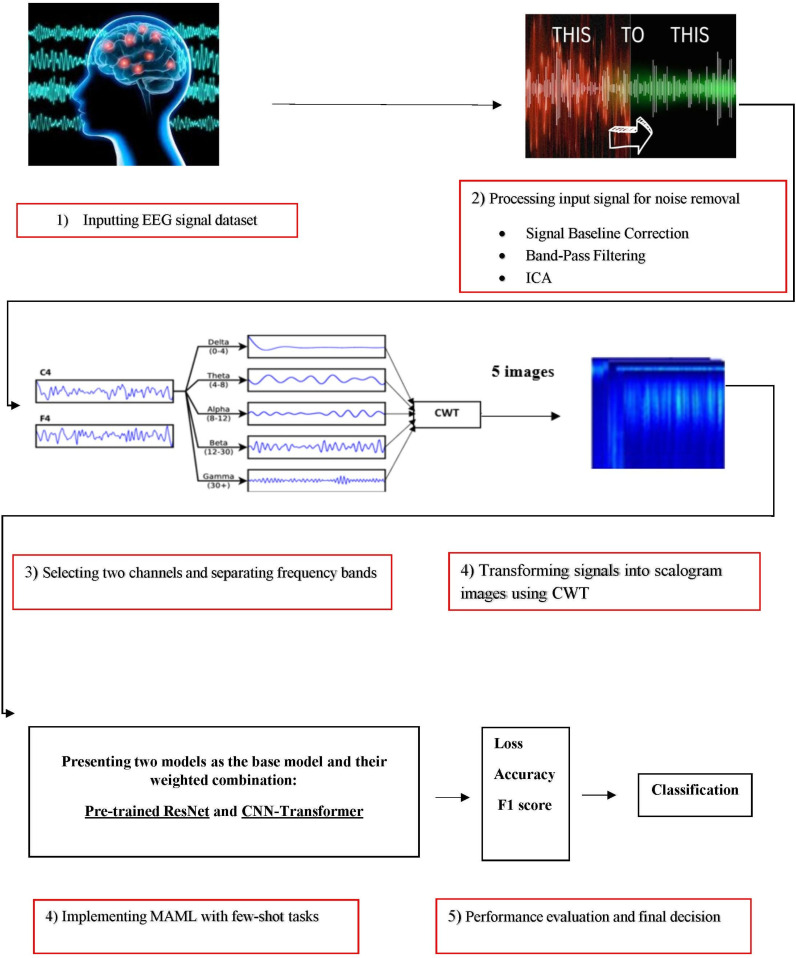
The main steps of our proposed method for PSD diagnosis.

The proposed approach is structured into five distinct phases. First, the EEG signals were pre-processed to ensure data quality. Next, relevant EEG channels were selected from the primary dataset to facilitate the identification of sleep deprivation. In the second phase, time-frequency images were generated from the processed EEG signals. The fourth phase focuses on developing a detection model for sleep disorders by designing an MAML algorithm tailored to few-shot tasks. Finally, we use standard evaluation metrics to assess the performance of the proposed FSL-based MAML model. An overview of the key steps in our method for diagnosing partial sleep deprivation is illustrated (Fig 1).

More details of the main steps are described in the following subsections.

### 2.1. Data acquisition

In this study, we used a secondary dataset sourced from [25]. A total of 26 participants, consisting of males and females aged between 22 and 36 years, who were in good health and free from any underlying medical conditions, were recruited for the study. EEG recordings were conducted on these 26 healthy individuals before and after experiencing Partial Sleep Deprivation (PSD). The participants were instructed to sit in a relaxed position with their eyes open while the EEG signals were captured using 31 electrodes. These electrodes were strategically placed on specific locations on the scalp based on the international 10–20 standard system, including sites such as Fp1, Fp2, Fz, F3, F4, F7, F8, Cz, C3, C4, T7, T8, Pz, P3, P4, O1, O2, Fpz, CPz, POz, AFz, FC1, FC2, FC6, FC5, CP1, CP2, CP5, CP6, P7, and P8. The EEG recordings were performed using a bandpass between 0.1 and 100 Hz and at a 250 Hz sampling rate.

#### 2.1.1. Ethics statement.

All experimental procedures were conducted following the principles embodied in the Declaration of Helsinki and were approved by the local Ethics Committee of Tarbiat Modares University, Faculty of Medical Sciences (approval no 1401.085; date of approval: June 6, 2022).

### 2.2. Data Pre-processing.

After data acquisition, the first step should be data preprocessing. We express some pre-processing steps that are usually done on EEG signals. The initial step involves conducting the signal baseline correction, followed by applying a notch filter to eliminate any power line artifacts. Subsequently, the EEG signal was subjected to band-pass filtering between 1 and 100 Hz, using a zero-phase linear finite impulse response filter. Lastly, the Independent Component Analysis (ICA) technique was employed using the “runica” method to effectively remove any undesired artifacts present in the signal, including EMG noises, ECG noises, eye blinks, or eye movements.

### 2.3. Transforming data

Due to the success of DL networks in image processing, we propose using frequency-time images gained by performing CWT on the preprocessed EEG signal in this study. Developing a sleep disorders diagnostic model with fewer channels can help reduce computational complexity. Therefore, we selected two electrodes, F4 and C4, from the most significant electrodes investigated in sleep research. Initially, the EEG signals were partitioned into five distinct bands: delta (<4 Hz), theta wave (4–8 Hz), alpha (8–14 Hz), beta (14–30 Hz), and gamma (30–50 Hz) [26]. Subsequently, frequency-time images were obtained for each frequency sub-band for the two electrodes, F4 and C4.

The Continuous wavelet transform (CWT) is a useful frequency-time analysis tool for non-stationary signals [27]. The CWT has the potency to develop a time series signal into the time and frequency domains. The CWT for a signal with translation parameter b, scale parameter a, and wavelet function Ψ(t) is as follows [28]:


$${CWT}_{f}^{\Psi }(a,b)=\int_{-\infty }^{\infty }f(tfrac1\sqrt{a}\Psi^{*}(\frac{t-b}{a})dt$$ (1)

The purpose of using a mother wavelet function Ψ is to locate a signal in both time and frequency. The CWT of a signal is a series of projections onto shifted and stretched kinds of Ψ(t) [29]. A two-dimensional image generated by CWT is called a scalogram image [30].

**Fig 2:**
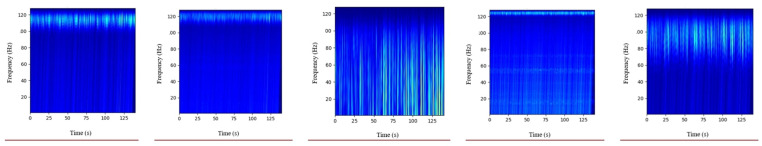
Frequency-time image obtained by the continuous wavelet transform.

Data in 2D format can extract more features than 1D data [12]. This research used the continuous wavelet transform to convert 1D EEG signals into 2D data. Initially, the raw 1D EEG signals underwent preprocessing using signal processing techniques. Subsequently, scalogram images were created through CWT to represent the 2D data. An example of the scalogram images derived from the EEG signals processed using CWT for different frequency bands is shown (Fig 2).

### 2.4. Designing a model based on few-shot learning-model-agnostic meta-learning

Designing models based on Few-Shot Learning (FSL) integrated with Model-Agnostic Meta-Learning (MAML) leverages the strengths of both FSL and MAML to enhance model performance, especially in scenarios with limited training data. By utilizing FSL, the model can effectively learn from just a few examples, which is essential when dealing with heterogeneous EEG datasets.

#### 2.4.1. Model Agnostic Meta-Learning.

MAML is an optimization-based meta-learning algorithm. MAML approximates the best parameter initialization for the base model that can rapidly adjust to new tasks in just one or a few steps of gradient descent. MAML can be used with any model that has been trained using gradient descent [30]. MAML consists of meta-training and meta-testing. Meta-training involves two primary optimization processes: the inner loop and the outer loop [31]. These processes are carried out on a group of related tasks during each iteration [32]. In the inner loop, the gradient on the support set of each task is computed to update the base model parameters to attain the updated parameters. Although these updated parameters are optimal for the specific task, they may not be optimal for multiple tasks. Therefore, the query set loss for each corresponding task is calculated by the outer loop after taking the updated parameters from the inner loop, and then gradient descent is executed to attain the optimal parameters for all tasks [33].

In meta-training, MAML learns a base model represented by a parameterized function $f_{\theta }$ with parameters θ over a set of training tasks $T=\left\{T_{1},T_{2},\ldots \right\}$. The task $T_{i}\;(in\;the\;N-way\;K-shots\;situation$ contains a training set $D_{i}^{tr}$ (also called support set) and a validation set $D_{i}^{val}$ (also called query set) [34]. In the inner loop, MAML changes the parameters θ to a task-adapted model parameter $\theta_{i}^{´}$ by gradient descent:


$$\theta_{i}^{´}\leftarrow \theta -\alpha \nabla_{\theta }L_{T_{i}}(f_{\theta };D_{i}^{tr}),$$ (2)

where α is the inner learning rate. In the outer loop of MAML, the model parameters denoted as θ are iteratively adjusted according to the loss incurred by the task-specific model on the validation dataset $D_{i}^{val}$:


$$\theta \leftarrow \theta -\beta \nabla_{\theta }\sum_{T_{i}\sim p(T)}L_{T_{i}}\left(f_{\theta_{i}^{´}};D_{i}^{val}\right),$$ (3)

where β is the outer learning rate [33].

For the implementation of the MAML model, it is crucial to select a suitable base network. The performance of MAML is greatly affected by the structure of the base network, as it serves as the initial parameter space and enables rapid learning for new tasks. The choice of architecture can significantly influence the model’s learning efficiency and ability to generalize. Various studies have utilized different base networks to implement MAML, each with unique characteristics. The following networks were used as the basic model in this study, along with an explanation of their structure:

- **Convolutional Neural Network-Transformer hybrid network**

This study suggests implementing a transformer-CNN architecture that integrates the strengths of transformers in modeling global long-range dependencies with the capabilities of CNNs to effectively capture spatial local semantic features [35]. Convolutional networks alone have weaknesses in capturing long-range dependencies and understanding the global context. To overcome this limitation, the CNN-transformer hybrid combines the strengths of both architectures. Convolutional layers efficiently extract low-level spatial features, while self-attention mechanisms enable the model to capture complex relationships across distant regions of the input.

The structure of our CNN-Transformer consists of three essential elements: the convolutional module, the Transformer encoder, and the classification head. The convolution module consists of two Conv2D layers with ReLU, two MaxPooling layers, a Batch Normalization layer, and a Dropout layer. These components are utilized for feature extraction, dimensionality reduction, normalization, and prevention of overfitting in convolutional neural networks.

The Transformer module consists of two Transformer Encoder layers. Each layer includes a Multi-Head Self-Attention mechanism to learn dependencies between features, an Add & Norm operation for stability and reducing dependence on data scale, a Feed Forward network for learning nonlinear features, and a second Add & Norm operation to improve gradient flow during training, enabling the model to process sequential data and better capture long-term relationships between features.

- **ResNet Network**

**Fig 3:**
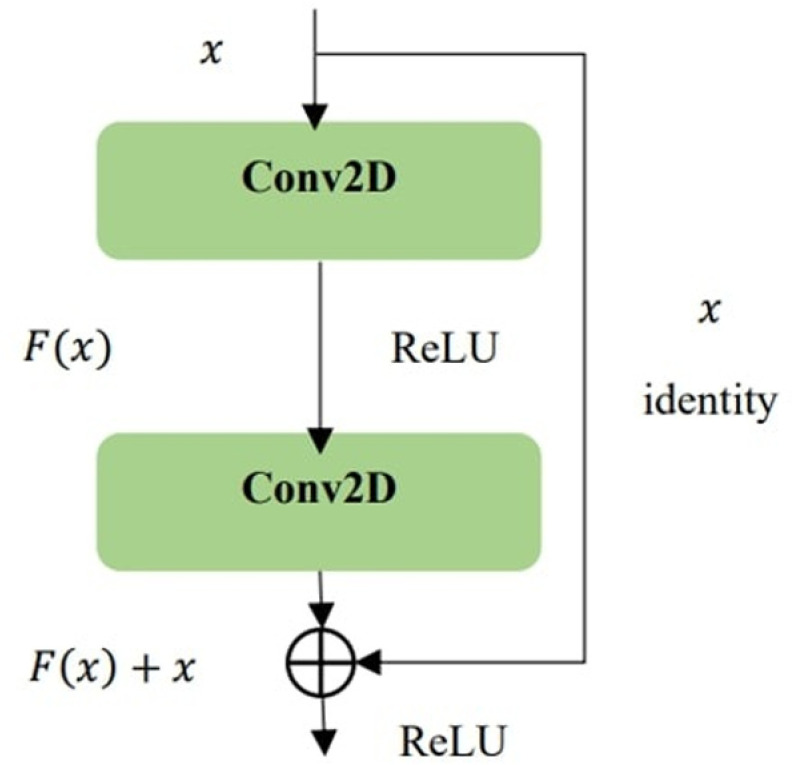
Residual block.

ResNet refers to a deep neural network architecture introduced by Kaiming He and his colleagues in 2015 at Microsoft Research Asia. The primary innovation of this design is the implementation of residual learning, which seeks to mitigate the challenges of gradient vanishing and performance decline that occur as the depth of the network increases during training. Conventional deep neural networks typically increase their depth by stacking multiple layers. However, as the number of layers grows, the gradients tend to diminish during backpropagation, complicating the training process. ResNet facilitates the learning of identity mappings through its residual learning framework, allowing the input to be directly passed to the output, thereby alleviating the issue of gradient vanishing. The fundamental component of ResNet is the residual block. A schematic representation of this block is illustrated (see Fig 3). Each residual block consists of two convolutional layers along with a skip connection. By directly adding the input x to the output of the final convolutional layer, ResNet ensures a direct flow of information and enhances the effective propagation of gradients [36]. ResNet-18 is composed of a convolutional layer followed by eight residual blocks. This architecture effectively addresses the challenges of vanishing or exploding gradients that can arise with increased depth in neural networks [37].

**• A hybrid of a CNN-Transformer and ResNet**

**Fig 4:**
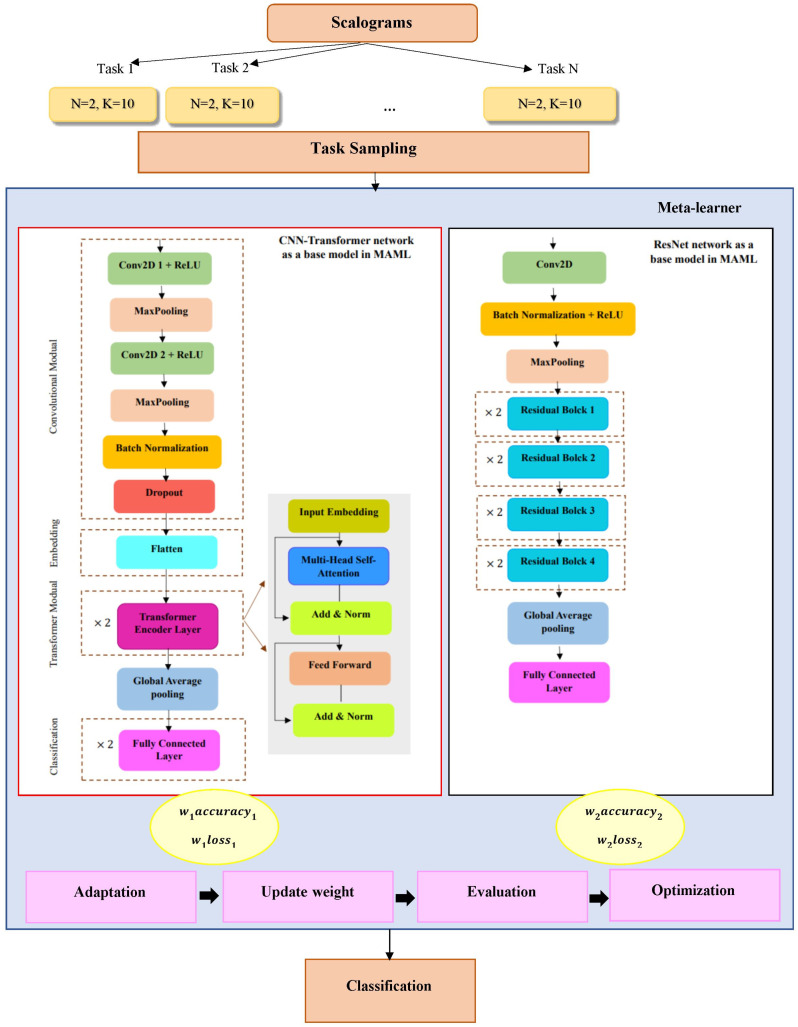
Schematic of the proposed Adaptive Weighted Dual MAML approach.

A challenge of MAML, like other meta-learning approaches, is its generalizability. This challenge arises for two reasons. One reason is that the meta-training tasks usually have a different distribution from the meta-test tasks. Another reason is that the number of tasks available in the meta-training phase is usually small (much less than the number of data available in conventional supervised learning), making meta-learning difficult to generalize [38]. Using a single base model in MAML may limit its ability to capture diverse task distributions and adapt effectively to new tasks. To address this, we leveraged two base models instead of one, allowing them to benefit from multiple perspectives during adaptation. Studies have shown that combining multiple classifiers improves performance compared to individual classifiers and predictors [39]. By assigning adaptive weights to the two base models, our approach dynamically balances their contributions, leading to improved generalization across varied tasks. This weighted hybrid model has been named Adaptive Weighted Dual MAML (Fig 4).

This approach offers several key advantages. Combining the two base models increases the diversity and comprehensiveness of the learned features, reducing the final model’s dependence on a specific learning path. Consequently, the performance fluctuations are minimized. The dual-architecture design further improves generalization by leveraging two distinct sets of features and gradients, which reduces the reliance on a single data distribution and leads to more stable performance on new tasks.

Additionally, dynamic weighting allows the model to adaptively select the optimal combination of the two base models for each task. This flexibility enables it to utilize the most relevant features from each model while avoiding excessive reliance on any single model, which may exhibit variable performance across different scenarios. A common challenge in meta-learning is sensitivity to initialization, which can lead to optimization instability across different tasks. By integrating the two base models, this approach functions as an adaptive averaging mechanism, mitigating sensitivity and improving optimization stability. Finally, while individual base models may be prone to overfitting on specific tasks, their combination with dynamic weighting helps prevent this issue, ensuring a more balanced and robust learning process.

### 2.5. Performance evaluation

Performance metrics are employed to evaluate the deep learning model’s ability for generalization. Accuracy indicates the percentage of correct predictions out of the total predictions made. On the other hand, the F1-score provides a more comprehensive evaluation by considering the model’s capability to accurately find true positives while minimizing false positives. The formulas for calculating the accuracy and F1-score are shown in equations 5 and 6. In these equations, TP, TN, FP, and FN represent true positives, true negatives, false positives, and false negatives, respectively [40].


$$Accuracy=\frac{TP+TN}{TP+TN+FP+FN}\;$$ (4)

$$F1-score=\frac{2TP}{2TP+FP+FN}\;$$ (5)

Additionally, to ensure the statistical reliability of the model comparisons, we employ 5-fold cross-validation combined with paired t-tests. This approach assesses the stability and consistency of the models’ performance across different data partitions and determines whether the observed differences between the models are statistically significant.

## 3. Results and discussion

Electroencephalogram (EEG) is a non-invasive and commonly utilized technique for diagnosing sleep disorders. As a time-varying non-stationary signal, EEG can benefit from the frequency-time analysis to improve diagnostic accuracy. The use of images derived from the frequency-time analysis is valuable in examining EEG signals [41]. In this study, the frequency-time images extracted from the EEG signals were initially trained using the CNN model, which is the most popular model in image analysis. The CNN model illustrated poor performance in PSD diagnosis. Therefore, we decided not to report its results. To improve the model’s performance, it is necessary to optimize the hyperparameters, change the architecture, and potentially increase the volume of data. The model’s poor performance may be due to insufficient data, preventing the model from identifying meaningful patterns. Since we aimed to reduce computing by utilizing fewer channels, the data augmentation solution was not suitable for us. Additionally, optimizing the architecture was assessed based on changes in the learning rate, number of neurons, and layers in the CNN. The CNN model proposed with these hyperparameters was the most suitable fit for our data, however, it exhibited poor performance.

After applying all of these settings, which did not yield satisfactory results, we concluded that there was a high probability that the poor performance of the CNN model may be due to the reason that deep learning models typically require large-scale and homogenous training data to develop a model with robust generalization ability. EEG signal datasets typically lack sufficient and homogeneous samples.

For the first time, this study utilized a model-agnostic meta-learning algorithm with multi-shot tasks, combining a CNN-Transformer hybrid and ResNet as the base model. This approach aimed to tackle the challenges linked to limited training data in EEG signal classification. MAML was chosen for its ability to facilitate efficient learning with minimal training examples, making it particularly suitable for scenarios where acquiring large-scale labeled EEG datasets is difficult or expensive. This algorithm enables the training of a meta-learner that can quickly adapt to new tasks with only a few examples, thereby enhancing the generalization capabilities of the model.

We attempted to improve the diagnostic capabilities of the proposed approach by adjusting the hyperparameters. Consequently, we examined how different hyperparameters affected the accuracy and loss of the classifier when utilizing MAML with few-shot tasks during the meta-training phase. The experimental results of the optimal hyperparameters can be found in Table 1. With a dataset of 416 images for training and 104 images for validation, and only 2 classes, the model can be trained with fewer tasks to learn and generalize. This speeds up the recognition of patterns and essential features. Therefore, a task number of 20 was chosen to ensure adequate accuracy and generalization for the model.

**Table 1:** Optimal hyperparameters of the model-agnostic meta-learning algorithm with few-shot tasks.

Hyperparameter	value
Number of shots	10
Number of tasks	20
Inner loop LR	0.001
Outer loop LR	0.001
Adaptation steps (Inner loop steps)	3

**Fig 5:**
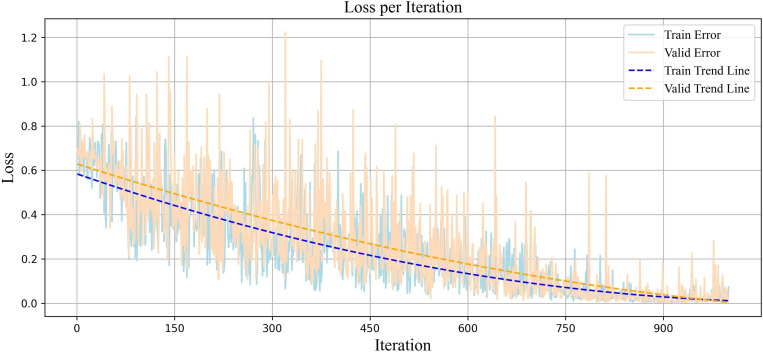
Plot of the loss function value per iteration for diagnosing partial sleep deprivation using the proposed Adaptive Weighted Dual MAML approach.

To assess the effectiveness of the suggested approach, a collection of performance measures such as loss function values, accuracy, and F1-score were utilized. The presented plots demonstrate the effectiveness of the proposed technique in the PSD classification. The proposed model achieved small and stable loss values for meta-training (Fig 5). The downward trend of the loss graph indicates gradual improvement during the training process. With each iteration, the error value decreased, demonstrating the model’s ability to identify patterns in the training data and make better decisions. Stabilizing the loss value after 900 iterations suggests that the model has reached a stable state during training.

**Fig 6:**
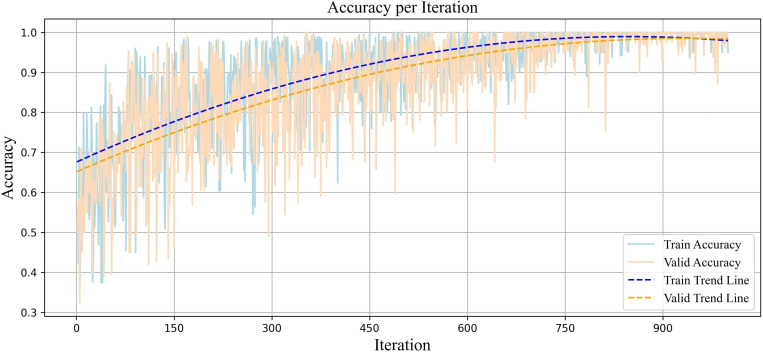
Plot of the accuracy per iteration for diagnosing partial sleep deprivation using the proposed Adaptive Weighted Dual MAML approach.

The accuracy plot across two datasets (training and validation) is illustrated (Fig 6). The upward trend of the accuracy graph shows continuous improvement in correctly detecting samples, with the number of correct predictions increasing. The stability of the accuracy at around 99% after 850 iterations indicates high performance. This stability indicates that the model has found a balance between learning and generalizability, with a strong ability to distinguish classes. The convergence and simultaneous increase in accuracy across both datasets indicate that the model not only has an effective structure for learning data but is also generalizable enough to avoid overfitting.

**Fig 7:**
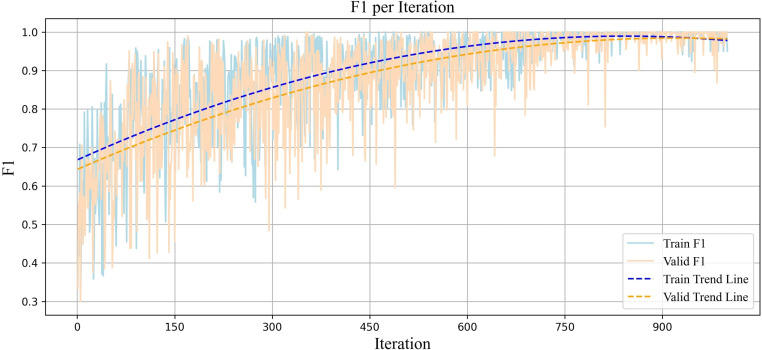
Plot of the F1-score per iteration for diagnosing partial sleep deprivation using the proposed Adaptive Weighted Dual MAML approach.

The F1-score plot for the training and validation datasets is illustrated (Fig 7). The upward trend observed in these curves reflects an improved balance between precision and recall, indicating the model’s strong and consistent performance in predicting classes.

In MAML, the model is not optimized for a specific task; instead, it is trained to perform well on any task with only a few updates. This diversity of tasks leads to fluctuations in the model’s performance, as each task presents its unique characteristics and challenges. As the model continuously faces new tasks and must quickly improve its ability to learn them, these changes result in momentary fluctuations in the evaluation measures. However, the overall trend is important, indicating the model’s success in gradually enhancing its generalizability to new tasks. This oscillatory behavior is expected and is an inherent feature of the MAML algorithm that enables the model to achieve optimal performance in new tasks with the minimal number of updates. In our approach, the model achieved a more stable and generalizable range across all evaluation criteria. This led to reduced fluctuations in performance across tasks compared to other models in our study that were designed with MAML and only had one base model.

To evaluate the model’s performance, two analytical tools were utilized: a confusion matrix and a t-SNE plot. This evaluation focused on the final 20% of the meta-tasks. This selection was made because, in meta-learning, the model progressively enhances its ability to recognize and differentiate between classes through successive meta-tasks, making the results from the concluding tasks a more reliable indicator of the model’s learning and consolidation.

**Fig 8:**
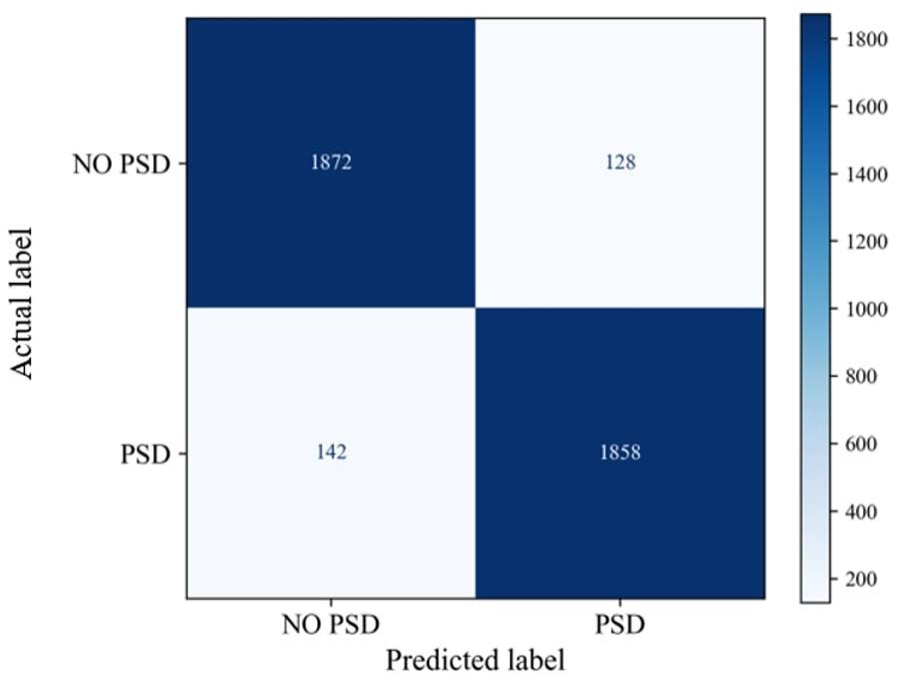
Confusion Matrix on the Last 20% Meta-Tasks.

The confusion matrix was constructed to visually compare the model’s predictions against the actual labels (Fig 8). The model accurately classified a substantial proportion of samples from each class, and the classification error rate remained within an acceptable range, reflecting satisfactory performance by the end of the meta-tasks.

**Fig 9:**
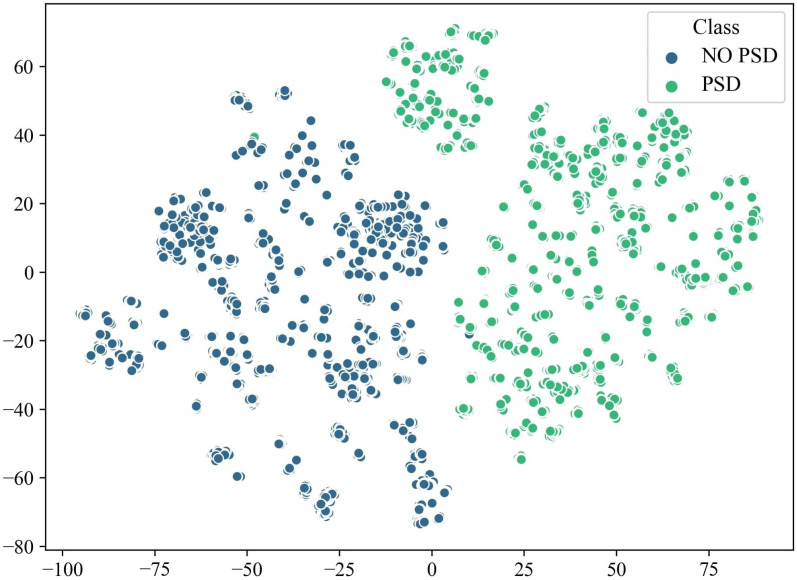
Visualizing data using t-distributed Stochastic Neighbor Embedding of the EEG Features Space for the Last 20% Meta-Tasks.

Additionally, to better understand the feature distribution and class separation within the feature space, a t-SNE plot was generated using the final 20% of meta-tasks (Fig 9). This two-dimensional projection of high-dimensional data enables visualization of relative clustering and class separability, confirming the model’s capability to distinguish between the two classes and demonstrating a well-structured, separable feature space at this stage.

Table 2 serves as an ablation study to evaluate the contribution of different base models within the hybrid MAML framework. All meta-learning settings, hyperparameters, dataset, and evaluation procedures were kept constant, and only the base learner was varied to assess its impact on the final performance. The results indicate that the CNN-Transformer-MAML model, while having a lower computational cost, delivers lower accuracy compared to the other two models. In contrast, the Adaptive Weighted Dual MAML effectively leverages the strengths of both base models, achieving a high accuracy of 99% on both the training and validation sets. This suggests superior generalization to new data compared to the standalone ResNet model. However, its high computational cost and extended execution time are notable limitations. Nevertheless, when accuracy is prioritized over computational efficiency, the Adaptive Weighted Dual MAML emerges as a highly reliable and effective choice.

**Table 2:** Comparison of performance and execution time for the designed models.

Model	Accuracy Train Validation		F1-score Train Validation		Execution time (Hour: Min)
CNN-Transformer-MAML	90%	88%	89%	88%	1:50 (Total time)
ResNet-MAML	99%	96%	97%	95%	4:45 (Total time)
Adaptive weighted dual MAML	99%	99%	99%	99%	4:45 (Total time)

The performance of the three meta-learning models—CNN-Transformer-MAML, Adaptive Weighted Dual MAML, and ResNet-MAML—was comprehensively evaluated using 5-fold Cross-Validation (CV), with accuracy and F1-score as the primary performance metrics, as presented in Table 3. Following cross-validation, paired t-tests were performed to statistically compare the models’ performance across the folds. The results indicated statistically significant differences between the models in all pairwise comparisons (p < 0.05). In particular, the Adaptive Weighted Dual MAML demonstrated significantly better performance compared to CNN-Transformer-MAML and ResNet-MAML. Regarding accuracy, the comparison between CNN-Transformer-MAML and Adaptive Weighted Dual MAML yielded a t-value of 9.51 (p = 0.0007), while the comparison between ResNet-MAML and Adaptive Weighted Dual MAML resulted in a t-value of 5.19 (p = 0.0065). Similarly, for the F1-score, Adaptive Weighted Dual MAML outperformed CNN-Transformer-MAML (t = 8.01, p = 0.0011) and ResNet-MAML (t = 4.06, p = 0.0150). These results underscore the efficacy of the adaptive weighting mechanism embedded within the MAML framework, suggesting that Adaptive Weighted Dual MAML provides a more robust and effective solution for meta-learning tasks compared to the alternative architectures examined in this study.

**Table 3:** Paired t-test Results from 5-Fold Cross-Validation for Performance Comparison of Meta-Learning Models Based on Accuracy and F1-Score.

Model’s name	CNN-Transformer-MAML	Adaptive weighted dual MAML		ResNet-MAML	CNN-Transformer-MAML	Adaptive weighted dual MAML		ResNet-MAML
Metrics	Accuracy	Accuracy		Accuracy	F1-Score	F1-Score		F1-Score
Fold 1	85	93		90	85	94		91
Fold 2	87	95		91	88	95		90
Fold 3	86	91		89	86	91		89
Fold 4	88	94		92	84	93		92
Fold 5	90	92		88	87	92		90
Paired t-test	t = 9.51 p = 0.0007		t = 5.19 p = 0.0065		t = 8.01 p = 0.0011		t = 4.06 p = 0.0150	

Previous studies have shown that classifiers based on ensemble learning, Support Vector Machines (SVMs), and K-Nearest Neighbors (KNNs) emerged as the most frequently utilized machine learning techniques for sleep analysis. These methods have shown a comparatively high average classification accuracy for the sleep analysis. However, the limitations of these classifiers are evident, particularly in their inadequate generalization of the developed models. Many of these approaches rely on a priori knowledge and manually extracted features, which is a labor-intensive process and challenging to implement in clinical trials. As a result, many researchers have shifted their focus toward deep learning, which has been widely applied across various domains [42,43].

Table 4 lists several studies conducted to automate the diagnosis of common sleep disorders using EEG and ECG signals, which are commonly used. This table shows the increased interest in analyzing sleep studies using deep learning methods in recent years. Another notable point evident from the table is that sleep deprivation has received less attention compared to more common sleep disorders such as sleep apnea and insomnia. While apnea and insomnia have been extensively studied and much research has been dedicated to developing automated methods for their detection, research on the automated detection of sleep deprivation is very limited. This is despite the fact that sleep deprivation can have profound and widespread effects on an individual’s physical and mental health. The insufficient research in this area highlights a significant scientific gap that could present significant opportunities for future research.

**Table 4:** Characteristics of studies that developed models for the automated diagnosis of sleep disorders.

First Author, Year	Disorders name	Dataset	Type of Models	Model Performance (Accuracy)
Hassan, 2015 [44]	Sleep Apnea	ECG	KNN	69.72%
Hassan, 2017 [45]	Sleep Apnea	ECG	SVM	75%
Wang, 2023 [46]	Narcolepsy	PSG EEG EEG + EOG	CNN CNN	78.94% 79.71%
Hassan, 2015 [47]	Sleep Apnea	ECG	Ensemble learning	80.07%
Hassan, 2017 [48]	Sleep Apnea	ECG	Ensemble learning	82.27%
Sharan, 2020 [47]	Sleep Apnea	ECG	CNN	88.23%
Qu, 2021 [48]	Insomnia	EEG EEG	CNN + RNN	90% 91%
Qin, 2022 [49]	Sleep Apnea	ECG	CNN + RLN, BiGRU-TDM	91.10%
Ivanko, 2020 [50]	Sleep Apnea	ECG	KNN	94.70%
Cooray, 2019 [51]	REM behavior disorder	PSG	Ensemble learning	96.50%
Ivanko, 2020 [40]	Sleep Apnea	ECG	SVM	98.70%
Our model	Partial Sleep Deprivation	EEG	Adaptive Weighted Dual MAML	99%

Sleep deprivation is a serious public health problem that has widespread negative effects on physical, mental, and cognitive health. It is associated with an increased risk of cardiovascular disease, diabetes, depression, decreased productivity, and even serious accidents at work or driving. However, traditional methods for diagnosing sleep disorders are usually time-consuming, expensive, and limited to specific settings such as sleep laboratories. These limitations indicate the need to develop automated tools for more accurate, faster, and accessible diagnosis of sleep deprivation. The present study aims to address this scientific gap by providing a solution based on new technologies such as machine learning and wearable devices. This approach can not only enable continuous monitoring and early detection of sleep problems but also contribute to reducing healthcare costs, preventing long-term complications, and improving the quality of life of individuals.

This study is the first to utilize meta-learning techniques for the classification of sleep disorders, marking a significant advancement in this field. To date, almost all previous research has been restricted to traditional machine learning methods or conventional deep learning approaches. This underscores a research gap in harnessing advanced techniques like meta-learning, which can greatly improve the accuracy and performance of diagnostic systems. The results of this study signify a crucial step toward broadening the utilization of state-of-the-art techniques in biomedical sciences and digital health.

We propose a hybrid base model based on the MAML algorithm with few-shot tasks for classifying the PSD. The MAML algorithm has previously been used in other areas of sleep analysis for sleep staging [7,52]. However, this study is the first to apply this method in the diagnosis of sleep disorders, specifically in partial sleep deprivation. We utilized only the F4 and C4 two channels of the EEG signals to analyze a person’s sleep situation, reducing the computational and hardware costs. These channels are the most effective for detecting sleep disorders, according to research. Due to the significance of the frequency sub-bands, we initially divided the EEG signals into five sub-bands: delta, theta, alpha, beta, and gamma. Next, the frequency-time images were obtained using CWT and were then utilized to train the Adaptive Weighted Dual MAML for the classification of PSD. Because the time-frequency images were obtained from small-scale and heterogeneous EEG signals, we proposed a model-agnostic meta-learning algorithm to classify them. In addition, we suggested a hybrid base model to improve the generalizability of the FSL-based MAML algorithm.

The results indicate that the evaluation criteria of the Adaptive Weighted Dual MAML model are superior to those of other models, particularly on the validation data. This enhancement highlights the effectiveness of combining the two base models in the MAML algorithm to improve the generalization and robustness of the sleep disorder diagnosis systems. As a groundbreaking study in this field, our research will serve as the basis for future exploration of meta-learning applications in sleep medicine.

Consequently, the use of an Adaptive Weighted Dual MAML model ensures strong generalization ability and high accuracy, offering a significant advancement in automated sleep disorder diagnosis. Therefore, the model proposed in this paper can serve as a beneficial decision support system for assisting clinicians. The characteristics of this decision support system include simplicity, speed, strong generalization ability, accuracy, and effectiveness in diagnosing PSD. The simplicity and efficiency of the system, due to its reliance on only two EEG channels, make it a viable candidate for real-time or wearable applications.

To address the limitations and enhance the applicability of the proposed model, several avenues for future research are recommended. First, future studies should involve a broader range of participants, encompassing various age groups, as EEG patterns and sleep-related brain activity are known to vary significantly with age. Expanding the demographic diversity of the dataset would improve the external validity and generalizability of the findings.

Secondly, this study focused on partial sleep deprivation as the target condition, enabling a detailed investigation within a controlled environment. However, this limitation may hinder the immediate application of the proposed model to other common sleep disorders. It is anticipated that the proposed framework will be valuable for understanding and addressing a wider range of sleep disorders, such as obstructive sleep apnea, insomnia, and narcolepsy. It is therefore anticipated that the outcomes of this study will provide a foundational basis for future research aiming to extend these findings to a broader range of sleep-related disorders.

Additionally, it is recommended to assess the model’s versatility and reliability across various datasets by re-evaluating the framework using different datasets with varying sizes and characteristics. Multi-task learning offers a promising approach to tackle challenges related to small-scale and heterogeneous EEG data. In our future work, we intend to incorporate multi-task learning on frequency-time images created from EEG signals for diagnosing several sleep disorders

Another important direction involves exploring the impact of using additional or alternative EEG electrodes beyond the two channels (F4 and C4) employed in this study. Although previous research has demonstrated the effectiveness of these electrodes in detecting changes associated with sleep deprivation, relying on a limited number of channels may result in missing valuable information from other brain regions. Future investigations should examine various electrode configurations to identify optimal setups that could improve the model’s performance and enhance its applicability in clinical and real-world environments.

Finally, considering the relatively high computational burden of the current deep learning model, future work should focus on optimizing the model’s architecture, applying pruning and quantization techniques, and exploring more efficient hardware platforms to enhance the processing speed. These improvements would be essential for enabling the model’s application in real-time and portable EEG monitoring systems.

## 4. Conclusion

In this study, we introduced an innovative methodology that utilizes the Model-Agnostic Meta-Learning (MAML) algorithm with few-shot tasks to improve the accuracy of diagnosing partial sleep deprivation (PSD) from small-scale and heterogeneous EEG samples.

We developed a weighted hybrid model by combining two base models, a CNN-Transformer and ResNet, within the FSL-based MAML framework, significantly improving the EEG signal classification performance. Traditional feature extraction methods from EEG signals often require substantial computational resources and expertise. To address this limitation, we employed frequency-time imaging, a method that converts one-dimensional signals into two-dimensional representations, thereby streamlining the feature extraction process. By utilizing the Continuous Wavelet Transform (CWT) for the frequency-time representation from only two EEG channels, F4 and C4, we successfully classified the EEG signals for the PSD diagnosis. Our results show that the FSL-based MAML method, combining two base models, achieves an average classification accuracy of 99% and an F1 score of 99%. Additionally, statistical analysis using 5-fold cross-validation and paired t-tests indicates that our proposed model significantly outperforms the two competing models.

This automated model has the potential to assist in the diagnosis and treatment of various sleep disorders by analyzing electroencephalographic (EEG) signals. Additionally, it serves as a valuable tool for brain-computer interface (BCI) technology, enabling direct communication between the brain and electronic devices, ultimately enhancing sleep quality and overall quality of life.
